# A Lightweight Radio Propagation Model for Vehicular Communication in Road Tunnels

**DOI:** 10.1371/journal.pone.0152727

**Published:** 2016-03-31

**Authors:** Muhammad Ahsan Qureshi, Rafidah Md Noor, Azra Shamim, Shahaboddin Shamshirband, Kim-Kwang Raymond Choo

**Affiliations:** 1 Faculty of Computer Science and Information Technology, University of Malaya, Kuala Lumpur, Malaysia; 2 Department of Computer Science, COMSATS Institute of Information Technology, Islamabad, Pakistan; 3 School of Information Technology & Mathematical Sciences, University of South Australia, Adelaide, Australia; Beihang University, CHINA

## Abstract

Radio propagation models (RPMs) are generally employed in Vehicular Ad Hoc Networks (VANETs) to predict path loss in multiple operating environments (e.g. modern road infrastructure such as flyovers, underpasses and road tunnels). For example, different RPMs have been developed to predict propagation behaviour in road tunnels. However, most existing RPMs for road tunnels are computationally complex and are based on field measurements in frequency band not suitable for VANET deployment. Furthermore, in tunnel applications, consequences of moving radio obstacles, such as large buses and delivery trucks, are generally not considered in existing RPMs. This paper proposes a computationally inexpensive RPM with minimal set of parameters to predict path loss in an acceptable range for road tunnels. The proposed RPM utilizes geometric properties of the tunnel, such as height and width along with the distance between sender and receiver, to predict the path loss. The proposed RPM also considers the additional attenuation caused by the moving radio obstacles in road tunnels, while requiring a negligible overhead in terms of computational complexity. To demonstrate the utility of our proposed RPM, we conduct a comparative summary and evaluate its performance. Specifically, an extensive data gathering campaign is carried out in order to evaluate the proposed RPM. The field measurements use the 5 GHz frequency band, which is suitable for vehicular communication. The results demonstrate that a close match exists between the predicted values and measured values of path loss. In particular, an average accuracy of 94% is found with R^2^ = 0.86.

## Introduction

Vehicular Ad Hoc Networks (VANETs) have been deployed in a wide range of real-world applications (e.g. safety [1], navigation [2], emergency healthcare [3], cooperative driving [4, 5], and infotainment services [6]), providing vehicle to vehicle (V2V) and vehicle to infrastructure (V2I) communications [7, 8]. Modern vehicular features, such as cruise control, also require V2V and V2I communications [9–11]. Thus, it is essential that VANETs are able to handle highly dynamic conditions, such as abruptly changing road topology, variable traffic density and different speeds of vehicles, efficiently in real-time. IEEE 802.11p standards are widely adopted in VANETs that operate at 75 MHz of spectrum in 5.9 GHz frequency band to facilitate V2X (both V2V and V2I) communications for dedicated short range communication (DSRC) [12, 13]. DSRC provides high data transfer rates to support VANETs applications in both V2V and V2I communications. The modern transport infrastructure comprises road infrastructural units, such as complex interchanges, flyovers, underpasses and road tunnels. Radio signal propagation is distinctively affected by each of the road infrastructure units utilized by a vehicle while communicating. For instance, road infrastructure unit, such as flyover, serves as a static radio obstacle that can potentially impede radio signals [14]. Moreover, radio propagation behaviour is likely to vary when deployed in restricted environments (e.g. tunnels) and free space [15].

The radio signals in VANETs can potentially be obstructed by different radio obstacles. The wavelength of the radio signals in 5.9 GHz frequency band is approximately 5 cm; therefore, they have relatively less penetrating power in comparison to technologies such as GSM that typically operates in 1800 MHz frequency band. In other words, radio signals in the 5.9 GHz frequency band are obstructed by static objects (e.g. buildings, dense vegetation, and advertising boards) and moving objects (e.g. large buses, trailers and delivery trucks that impede radio signals) present in the VANETs environment [16, 17]. Thus, vehicular communication built on IEEE 802.11p standards suffers from a relatively small effective coverage communication area, and potential disruption that results in signal attenuation (e.g. due to radio obstacles).

The simulation provides a cost effective solution to evaluate new protocols and applications. The mobility models have to be implemented prior to the evaluation of the protocols [18–20]. Likewise, the radio propagation models (RPMs) in VANETs are designed to accurately predict path loss, and the choice of RPM affects the ultimate performance of a particular application in VANETs. An effective RPM is one that is simple and computationally inexpensive to provide accurate prediction of path loss while addressing a range of radio obstacles and other requirements in modern road infrastructure [21]. Existing RPMs, such as [15, 22–24], allow the prediction of path loss in road tunnels. However, these RPMs neither take the field measurements in a frequency band suitable for VANETs deployment nor provide one or more critical features, such as modelling of moving obstacles. This is the gap that we attempt to address in this paper.

In this paper, we propose a computationally inexpensive radio propagation model suitable for vehicular communication in road tunnels. The field measurements of signal attenuation are taken at 5 GHz; therefore, the results are equally applicable to predict the radio propagation properties at 5.9 GHz. We utilize the basic geometric properties of a road tunnel to obtain a relationship between the geometric properties of the tunnel and signal attenuation. Additional signal attenuation caused by the moving radio obstacles in tunnels is also considered in the proposed RPM. Finally, the proposed RPM is validated using data obtained from an extensive field measurement campaign. A comparative summary is also presented in this work.

The remainder of the paper is organized as follows. Section 2 presents related work and background materials. Section 3 presents the proposed RPM suitable for road tunnels in VANETs deployment. Section 4 describes the experimental setup and findings. Section 5 presents the adherence of the proposed model with the measured values of signal attenuation. The comparative summary is detailed in Section 6. Section 7 concludes the paper.

## Related Work

A significant amount of efforts have been devoted to designing effective RPM in VANETs. The modelling approach of radio propagation in VANETs can be broadly categorized into direct, microscopic and macroscopic. In direct approach, quantities, such as received signal strength (RSS) and signal attenuation, are empirically measured at a predefined physical site. The direct approach is simple but labour intensive, and the physical factors that constitute the measured results remain uncertain. [25, 26]. In the microscopic approach, individual entities along with the quantities of interest (e.g. individual position and speed of vehicle at a particular time) are considered to approximate radio propagation [27]. The microscopic approach is computationally expensive as individual quantities are considered for each entity in the vehicular environment. In the macroscopic approach, gross quantities, such as average speed of vehicle, are considered and simple geometrical concepts are applied to model radio propagation; thus, such an approach is computationally less complex [28]. However, macroscopic approach is not considered realistic due to the inclusion of gross quantities.

Another classification of RPMs is expressed in [29], where RPMs are categorized to be either empirical or deterministic. The former contains mathematical expressions with the parametric definitions of environmental characteristics and communication system properties. The accurate prediction of signal attenuation from an empirical RPM depends upon the similarity of the model parameters and actual physical environment. In deterministic RPMs for tunnels, physical factors affecting radio propagation, such as reflection, diffraction and Doppler’s effect, are utilized to determine the path loss. Deterministic RPMs may use numerical methods to solve Maxwell equations [30, 31], ray tracing approach [32, 33], and the tunnel as waveguide that utilize waveguide properties [34]. However, the computational complexity is a major drawback of deterministic RPMs for tunnels.

In VANETs simulations, basic RPMs, such as free space model [35] and two ray ground [36] models, are widely adopted. However, these generally do not consider modern road infrastructure units in the prediction of path loss. CORNER [16] is a propagation model of urban vehicular networks, which utilizes a path loss and attenuation formula defined in [37]. This formula requires the exact position of vehicles relative to the urban street grid. The three possible conditions of LOS, NLOS1 and NLOS2 are considered in this study. In NLOS1 condition, two communicating vehicles are separated by one corner, while in NLOS2; communicating vehicles are separated by two corners. CORNER uses a three-step process for the prediction of signal attenuation, namely: 1) a path traversed by the radio signal between sending and receiving vehicles is determined, 2) the LOS condition is identified between the communicating vehicles, and 3) the propagation formula is applied. The analysis is performed on the collected data using connectivity index, average hops, average node degree, and average link duration. CORNER is based on urban road infrastructures having streets and junctions, but does not consider individual vehicles as potential radio obstacles.

A tunnel can be considered a waveguide due to its geometry and the conductivity; therefore, radio propagation in tunnels can be modelled using waveguide properties. According to the literature, transverse dimensional tunnels are much larger than radio signal’s wavelength experience waveguide properties [34, 37]. Therefore, signal attenuation in tunnels is smaller as compared to signal attenuation in free space because of the waveguide effect. However, the waveguide model is only suitable for calculating accurate signal attenuation in straight tunnels.

Two-slope propagation models for tunnels are empirical propagation models based on two-ray ground model suitable for LOS conditions among communicating vehicles. The path loss curve is divided into near and far regions in two-slope propagation models. In the near region, there is a rapid decrease in path loss slope usually modelled as free space propagation; while the path loss slope is reduced in the far region due to waveguide effect. The point where the near and far regions can be separated from each other is called break point. The location of the break point depends on the wavelength of the radio signal, the dimensions of the tunnel, the antenna, and the relative permittivity [38]. However, the exact identification of break point is only determined experimentally.

The parameters that affect the radio propagation in tunnels include tunnel geometry, electromagnetic properties of the material used to build the tunnel, antenna characteristics, and radio obstacles [29]. Earlier research on radio propagation inside tunnels has shown that the dimensions of tunnels have a significant impact on the signal attenuation [23, 39, 40]. However, the influence of conductivity on the radio propagation can be neglected in most tunnels [41]. Moreover, the signal attenuation inside the tunnels depends on radiation pattern, polarization and position of transmuting and receiving antennas [38, 42–44].

Moving radio obstacles in the tunnel cause additional signal attenuation. An earlier study [24], for example, showed that the additional path loss in tunnels caused by moving radio obstacles can reach around 50 dB for 900 MHz frequency band. Another study [23] showed that the additional path loss in VHF band caused by moving radio obstacles in tunnels depends predominantly on size and quantity of moving radio obstacles. Furthermore, the shape and dimension of the tunnel cross section (circular or rectangular) can impact on the rate of signal attenuation [29]. However, findings from [22] suggested that the rate of attenuation in UHF band is not dependent on the shape and dimension of the tunnel, if the tunnel cross section is 15 times larger than the wavelength of the radio signal.

From the existing literature, it is identified that several existing RPMs are computationally complex as they are deterministic and a large number of RPMs for tunnels have not been evaluated for deployment in a frequency band suitable for VANETs. Furthermore, most existing RPMs do not consider additional attenuation from large moving objects in road tunnels. Therefore, a lightweight RPM with minimal number of parameters for vehicular communication in road tunnels is required, particularly if it has been evaluated for suitability for VANETs deployment. This is the focus of this paper.

## Proposed RPM for Road Tunnels

This section describes our proposed RPM for road tunnels. The key design goal is namely: an RPM which utilizes a minimal set of parameters to estimate path loss in an acceptable range.

According to the literature, there is a steep decrease in the RSS values initially and as the distance between the communicating vehicles increases, RSS gradually deteriorates. The inverse correlation between RSS value and the distance between the communicating vehicles demands that the path loss in tunnel is logarithmically proportional to the distance between the communicating nodes as shown in Eq (1).

$$PL_{M}\left[dB\right]=k\;{log}_{10}\left(d\right)$$ (1)

In Eq (1), ***PL***_***M***_ denotes the major component of path loss, ***d*** is the distance between transmitting vehicle (Tx) and receiving vehicle (Tr), and ***k*** represents a constant of proportionality determined using physical properties of the road tunnel. The value of ***k*** also depends on radio signal characteristics, such as wavelength of the transmitted signal. The proposed formula for the calculation of ***k*** is shown in Eq (2), where **h**, ***w*** and ***λ*** represent the height of the road tunnel, the width of the tunnel, and the wavelength of the transmitted signal, respectively. In Eq (2), ***r*** is the difference of height and width of the road tunnel.

$$k=r+\frac{w}{h\lambda }$$ (2)

Eq (2) can be used to calculate ***k*** if the width ***w*** of the tunnel is greater than the height ***h***. If the height of the tunnel is greater than its width, then the value of ***k*** is calculated as $\left(r+\frac{h}{w\lambda }\right)$. The impact of moving radio obstacles on the radio propagation can be estimated using the single knife-edge model, as this model can be applied in situations where the wavelength of the radio signal is significantly smaller than the size of the radio obstacle [17]. Therefore, the single knife-edge model is well suited for the vehicular communication. An approximation of the additional path loss in dB caused by the moving radio obstacles in vehicular communication can be represented using Eq (3).

$$PL_{AM}\left[dB\right]=6.9+20{log}_{10}\left[\sqrt{{\left(\upsilon -0.1\right)}^{2}+1}+\upsilon -0.1\right]$$ (3)

where

$$\upsilon =\;\sqrt{2}\;\frac{H}{r_{f}}$$ (4)

In Eq (4), ***H*** denotes the difference of the height of the radio obstacle and the height of the straight line connecting the communicating vehicles. The presence of the radio obstacle within 60% of the first Fresnel’s zone ellipsoid is the cause of the additional attenuation. Therefore, Eq (4) includes a parameter ***r***_***f***_, which is the radius of the first Fresnel’s zone ellipsoid [17] and is obtained by Eq (5).

$$r_{f}=\;\sqrt{\frac{\lambda d_{obs}\left(d-\;d_{obs}\right)}{d}}$$ (5)

In Eq (5), ***d***_***obs***_ is the distance between the obstacle and Tx. The approximation for the total path loss is the combination of ***PL***_***M***_ and ***PL***_***A***_ given in Eq (6).

$$PL\left[dB\right]=\;PL_{M}\left[dB\right]+\;F\left(PL_{A}\left[dB\right]\right)$$ (6)

In Eq (6), ***F*** denotes the probability of the large moving obstacles to interrupt the line-of-sight (LOS) between communicating vehicles. The value of ***F*** is dependent on the ratio of moving radio obstacles to the total number of communicating vehicles.

The calculation of additional attenuation caused by the moving radio obstacles in simulation is the most computational expensive step if the microscopic view of the environment is considered, because at least an O(m log m + n) line intersection algorithm [45] is required to compute n intersections among m lines. However, in this paper, the maximum signal attenuation caused by moving radio obstacles is calculated only once. The probability ***F*** of the moving obstacles to interrupt LOS among communicating vehicles is multiplied with the maximum signal attenuation caused by moving obstacles in order to reasonably estimate the additional path loss. Therefore, the calculation of additional attenuation due to moving radio obstacles is an algorithm with a constant running time.

## Measurement Campaign

We carried out an extensive field measurement campaign for the sole purpose of validating the proposed RPM. It is to be noted that no specific permissions were required to carry out the field study at the location specified in experimental setup section. This is because the site chosen for the field measurement is a part of national highways and the communicating vehicles were driven within the legitimate speed limit. Furthermore, the field study did not involve any endangered or protected species.

### Experiment Setup

We carried out an extensive data gathering campaign in the Kohat tunnel (Khyber Pakhtunkhawa, Pakistan, latitude: 33.6570 longitude: 71.5420) using 802.11 n WiFi devices configured at 5 GHz. The Kohat tunnel is a fairly straight tunnel with measuring 1.9 Km in length, 10.3 m in width, and 6 m in height. The communicating nodes consist of an Intel Dual Band Wireless-N 7265 wireless adapter connected to a D-Link ANT70-0800 antenna, which provides a gain of 10 dBi at 5 Ghz. The antenna was connected with the wireless device using a cable of length 3 m and approximately 3 dB loss. We utilized a Garmin GPS 18x USB receiver to locate the position of nodes. The vehicles used in the experiments were a Honda City 2007 (height: 1495 mm) and a Suzuki Mehran (height: 1410 mm). The antenna and the GPS receiver were mounted on the vehicles’ roofs.

### Measurement Scenarios

Multiple experiments were conducted using different setups. In the simplest setup, a vehicle (Tx) was parked inside the tunnel and the RSS was measured using the other vehicle every 50 meters. At certain points during the field testing campaign, GPS signals were lost due to lack of inter-visibility with GPS satellite. Therefore, the vehicle tachometer was used manually to measure the distance among the communicating nodes on the points where no valid GPS signal was detected. In another scenario, the RSS is measured while Tx was stationary and vehicle (Tr) was driven at 40 km/hr and the test is repeated at 60 km/hr. In one of the setups, both vehicles were driven at variable speeds in such a manner that one vehicle approaches the other vehicle with greater speed and then leaves it behind for the rest of experiments.

Table 1 outlines the multiple setups.

**Table 1:** Measurement Setups.

Scenario	Tx Speed	Tr Speed	Updation	Orientation
*SC*_*1*_	Stationary	Stationary	50 meters	Tr going away from Tx
*SC*_*2*_	Stationary	40 km/hr	1 meter	Tr going away from Tx
*SC*_*3*_	Stationary	70 km/hr	1 meter	Tr going away from Tx
*SC*_*4*_	Stationary	54 km/hr	15 meters	Tr going away from Tx
*SC*_*5*_	Stationary	72 km/hr	20 meters	Tr going away from Tx
*SC*_*6*_	30 km/hr	60 km/hr	10 meters	Tr approaching Tx and then going away

### Findings

This subsection presents and discusses the findings obtained from the field measurement campaign. The communication performance was measured and analyzed under the different setups in order to identify factors resulting in major change in signal attenuation. By identifying such factors, we could fine-tune future measurements and omit factors that do not have a significant impact on path loss. The communication performance was analyzed using the measured RSS as a function of distance between the communicating nodes. In order to summarize the results, we utilized packet delivery ratio (PDR) along with path loss in dB as another performance indicator.

**Fig 1:**
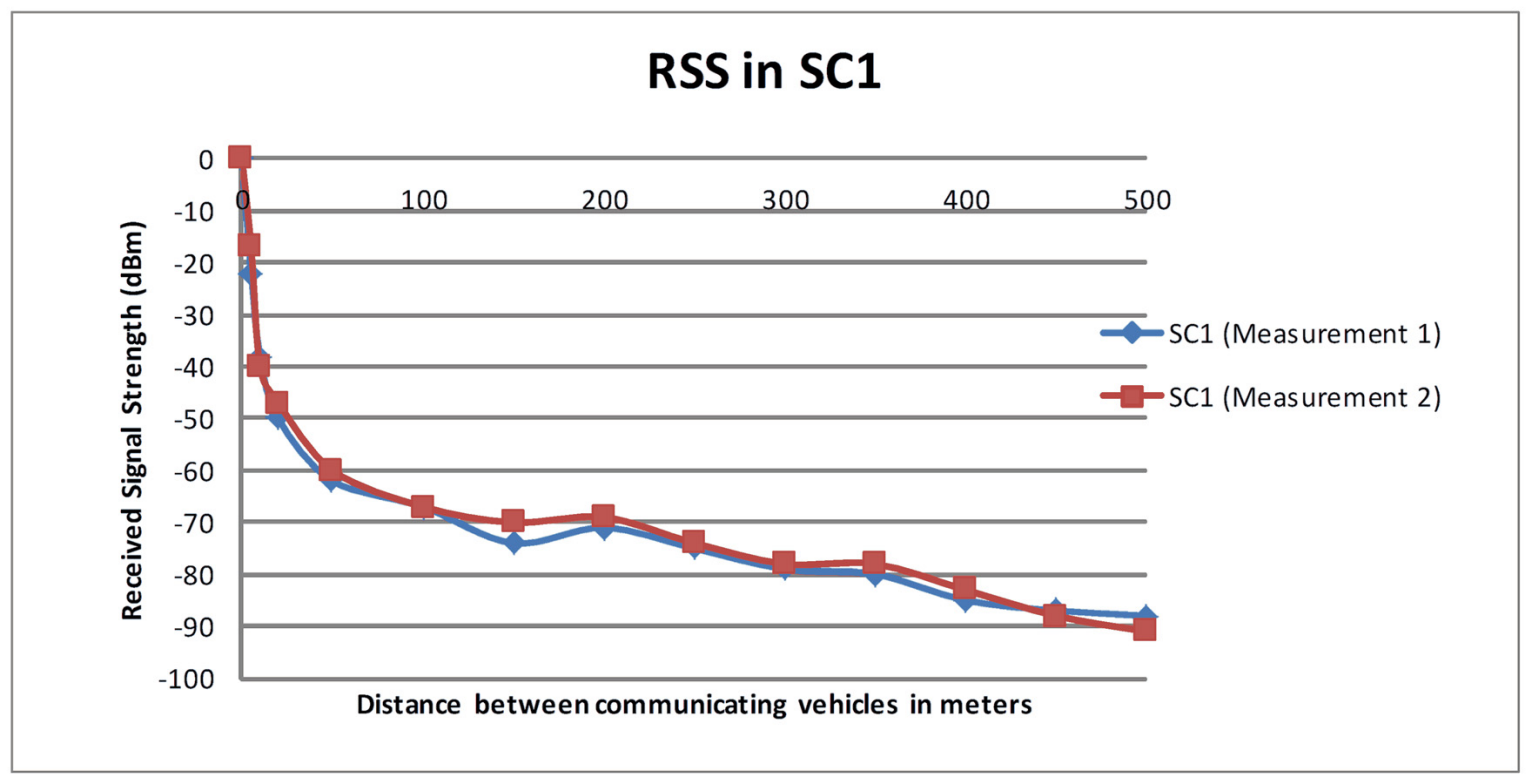
RSS in *SC*_*1*._

Fig 1 shows the RSS in dBm as measured in *SC*_*1*_. Due to lack of valid GPS signals in one of the measurements, we manually calculated the distance between the communicating nodes (measurement 1). However, the same test was repeated close to the entrance of the tunnel using GPS signals and no significant difference was found between the two measurements. Both measurements are shown in Fig 1. It is clearly visible that the radio signal quickly deteriorates when Tx is close to Tr. However, the decrease in the RSS is gradual when the Tr is more than 100 meters away from Tx.

**Fig 2:**
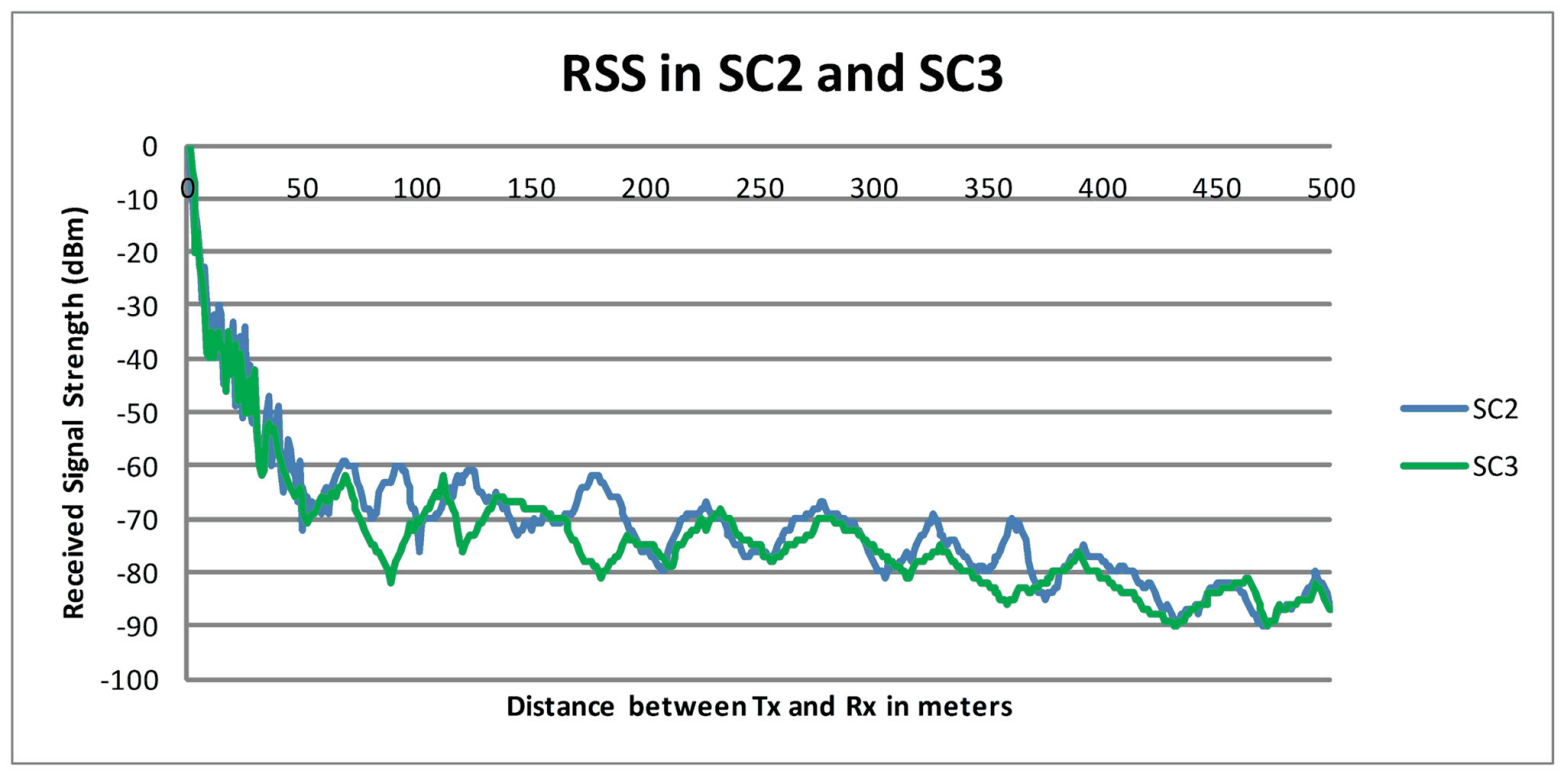
RSS in *SC*_*1*_ and *SC*_*2*._

Fig 2 presents the RSS in dBm as measured in *SC*_*1*_ and *SC*_*2*_. A sudden decrease in RSS is observed when Tr moved away from Tx during the first few meters. This decrease in RSS becomes gradual after about 50 meters. As the distance between the communicating nodes increases, the change in the signal strength became steady probably due to the waveguide effect. This steady signal strength can be observed until the distance among communicating nodes reach almost 350 meters. When the distance between the communicating vehicles exceeded 350 meters, a significant deterioration in the signal strength was observed. There is no major difference in signal attenuation in *SC*_*1*_ and *SC*_*2*_, which implies that the speed of the vehicle has very little or no effect on the signal attenuation in the maximum legal speed limit.

**Fig 3:**
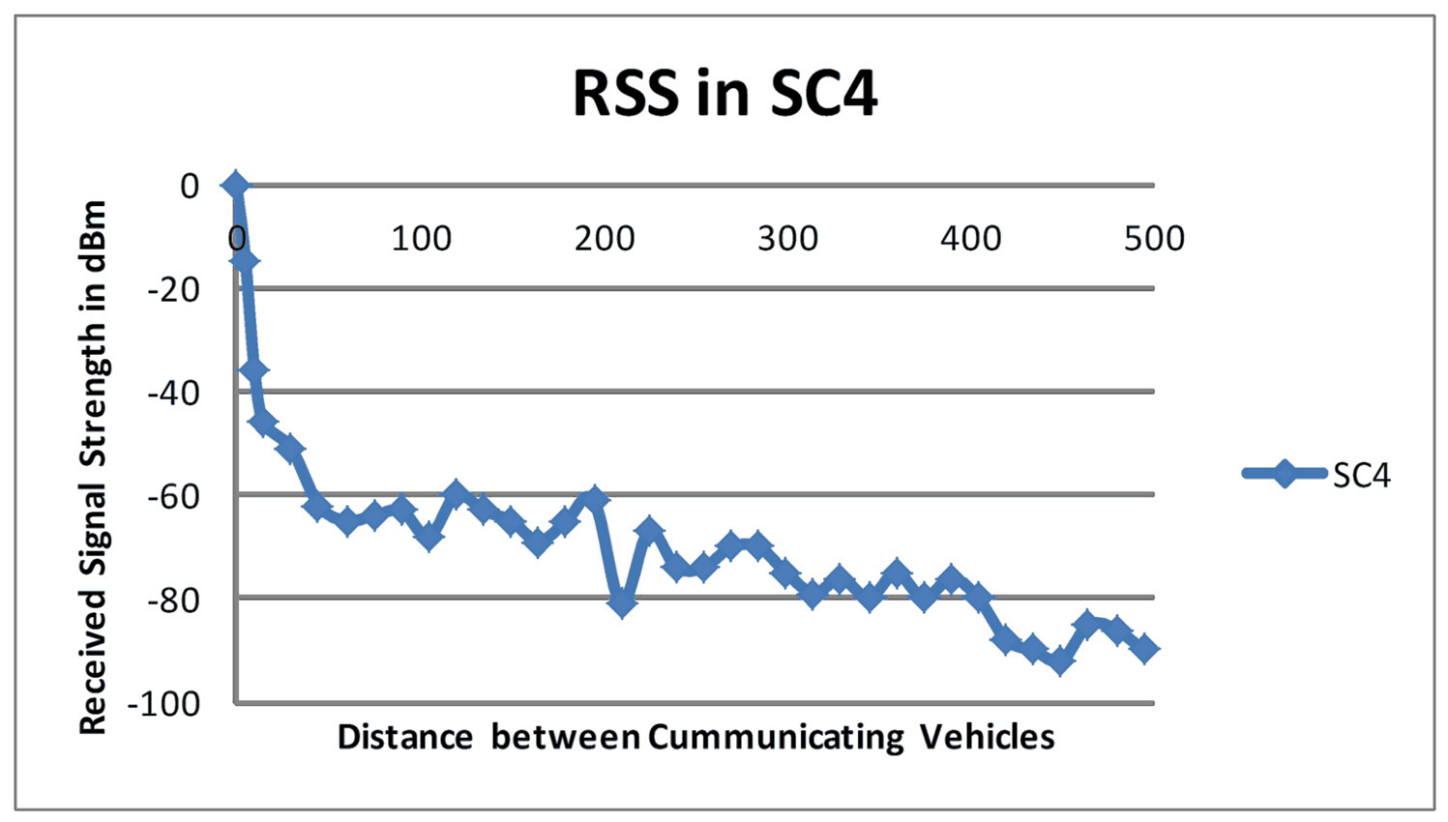
RSS in *SC*_*4*._

**Fig 4:**
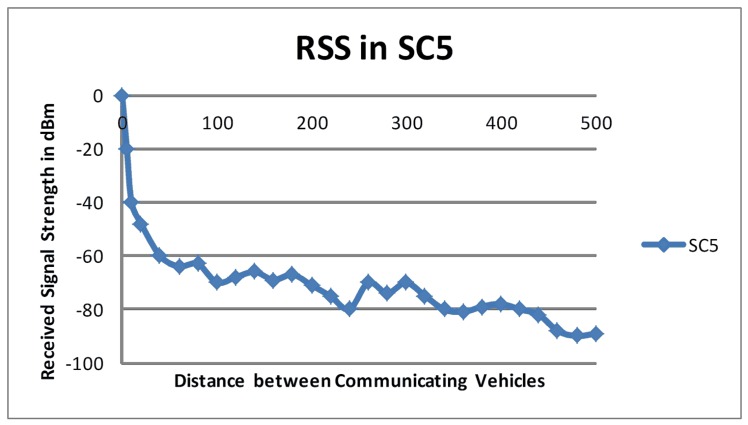
RSS in *SC*_*5*._

Figs 3 and 4 show the degradation in RSS for the scenarios *SC*_*4*_ and *SC*_*5*_, respectively. Tx was again parked and Tr was driven at 54 km/hr and 72 km/hr for *SC*_*4*_ and *SC*_*5*_, respectively. The measured RSS values in *SC*_*4*_ and *SC*_*5*_ supported the findings from *SC*_*2*_ and *SC*_*3*_ because a sudden deterioration in RSS was observed in the first few meters followed by a gradual decline in RSS. Although Tr was driven at different speeds in *SC*_*4*_ and *SC*_*5*_, there was no significant difference in the RSS values observed in these setups.

**Fig 5:**
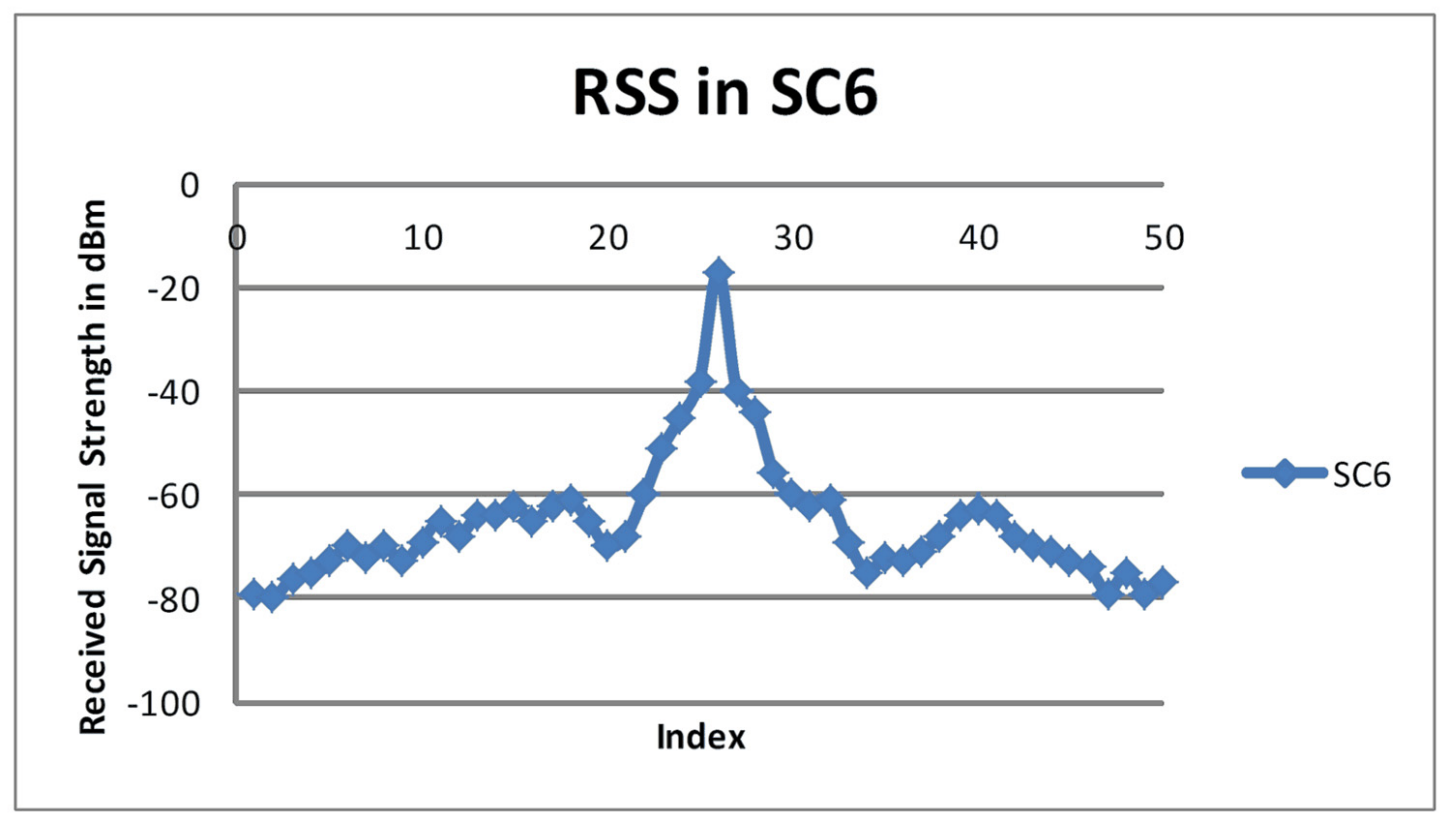
RSS in *SC*_*6*._

In *SC*_*6*_, both Tx and Tr were driven at variable speeds such that Tr was behind Tx at the start. The faster Tr approaches Tx in approximately 30 seconds and overtakes Tx. Fig 5 represents the measured RSS values in *SC*_*6*_. The RSS started to increase slowly as the distances between Tr and Tx decreased. The RSS increased as Tr approached Tx, but decreased as the distance between the communicating vehicles increased again due to the higher speed of Tr. The results obtained from *SC*_*6*_ also echoed findings from other setups where one vehicle was stationary.

### Effect of Moving Obstacles

It is observed from the measurement campaign that moving radio obstacles had a significant impact on radio propagation in road tunnels. Based on our findings and considering the actual road traffic situation, it is clear that large moving vehicles (e.g. delivery trucks) caused an additional decrease in RSS of about 20 dB to 30 dB. The additional signal attenuation due to moving radio obstacles is evident from the different setups, for instance, a decrease of about 20 dB was observed in *SC*_*3*_ as opposed to *SC*_*2*_ when the vehicles were about 170 meters apart. Therefore, moving radio obstacle appears to cause additional attenuation in road tunnels.

## Validation

This section explains the effectiveness of the proposed RPM for the prediction of path loss in vehicular communication.

**Fig 6:**
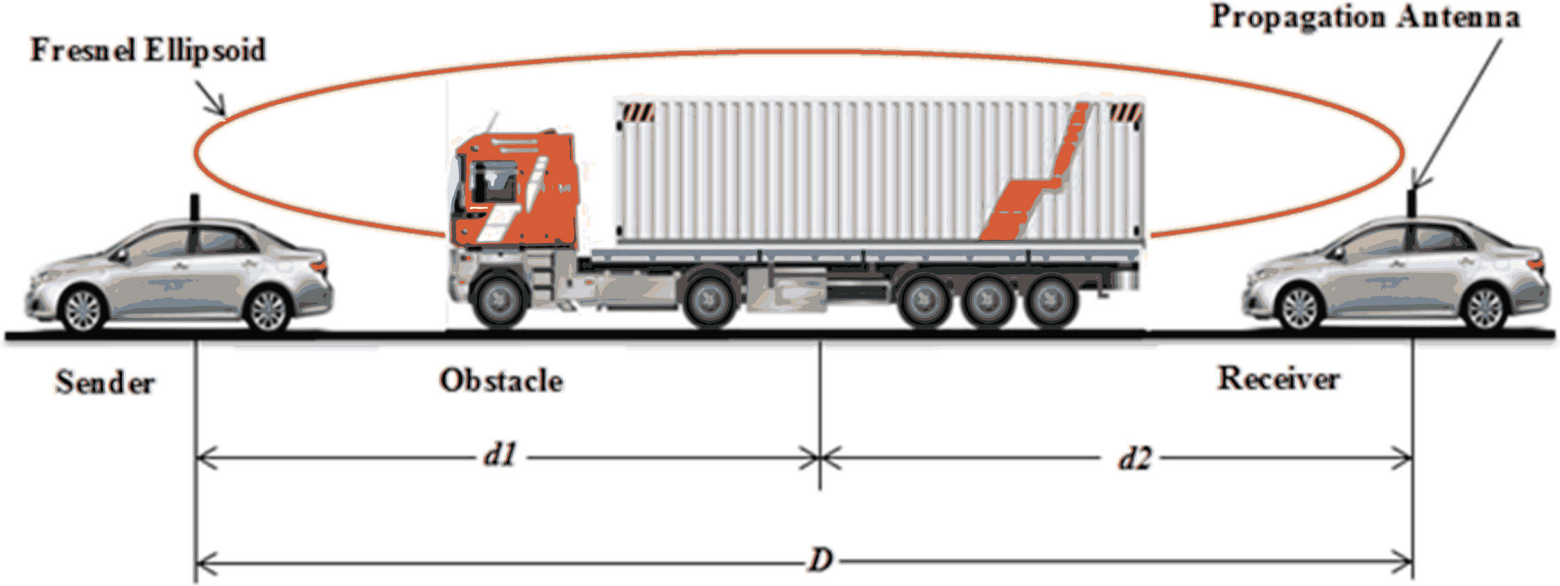
Fresnel’s Ellipsoid.

The proposed RPM for road tunnels was validated by comparing the predicted path loss with the data acquired during the measurement campaign. The RSS values obtained from the field measurement campaign in the different setups were converted to the path loss. The additional path loss ***PL***_***A***_ caused by the moving radio obstacle was calculated using Eq (3). We obtained a maximum value of path loss for the most common case, where Tx was close to the moving obstacle and more than 60% of the first Fresnel’s zone ellipsoid was obstructed by the moving obstacle. This scenario is shown in Fig 6.

The maximum value of the additional path loss ***PL***_***A***_ is found to be approximately 30 dB using Eq (3) for the most commonly occurring case. For the value of ***F***, we carefully examined the frequency of heavy traffic in the tunnel. As the measurement site was along a busy national highway, the frequency of heavy traffic was relatively high. The value of ***F*** is considered to be 0.2 to allow comparison between the predicted value of path loss and the actual measurement results.

**Fig 7:**
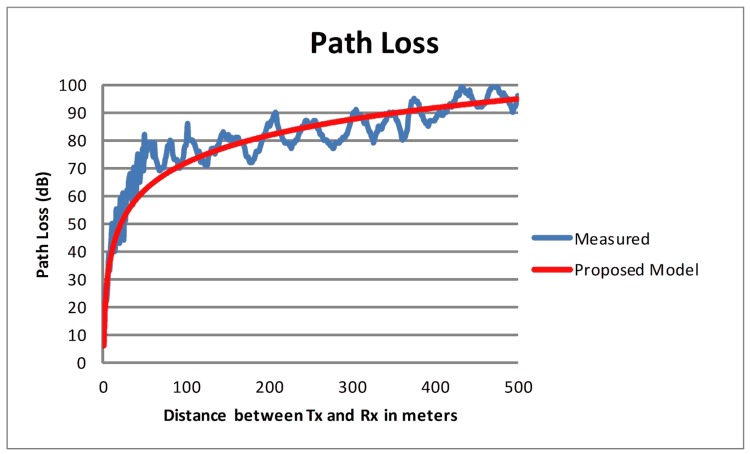
Path Loss (Measured vs Predicted).

Fig 7 shows a comparison of predicted path loss with the measured results.

The R^2^ value obtained by the statistical analysis yields 0.86, which confirms the applicability of the proposed RPM to predict path loss in road tunnels for vehicular communication. A residual analysis on the data was also performed and no clear pattern was observed. Furthermore, an average accuracy of 94% is found between the measured and predicted values of path loss. The R^2^ value, the residual analysis, and the average accuracy of the predicted path loss reaffirmed the suitability of the proposed model to predict path loss in road tunnels for the frequency band suitable in a VANET deployment.

## Discussion

The path loss in road tunnels is relatively low as compared to other road infrastructure units probably due to waveguide effect (see Section 2). Therefore, the free space model over-estimates the path loss in a road tunnel similar to the situation with CORNER, which considers an LOS scenario to be modelled as free space propagation. Hence, employment of both free space model and CORNER yields the same values of path loss which are not suitable for predicting path loss in road tunnels. However, the two-ray ground model does not over-estimate the path loss when the transmitter and the receiver are close to each other (approximately within 60 meters). The prediction of path loss from the two-ray ground model gradually starts to “exaggerate” as distances between the communicating vehicles increase, until it coincides with the free-space model and CONER. Therefore, all three models (i.e. free-space model, two-ray ground model and CORNER) are not suitable to predict path loss in a road tunnel.

**Fig 8:**
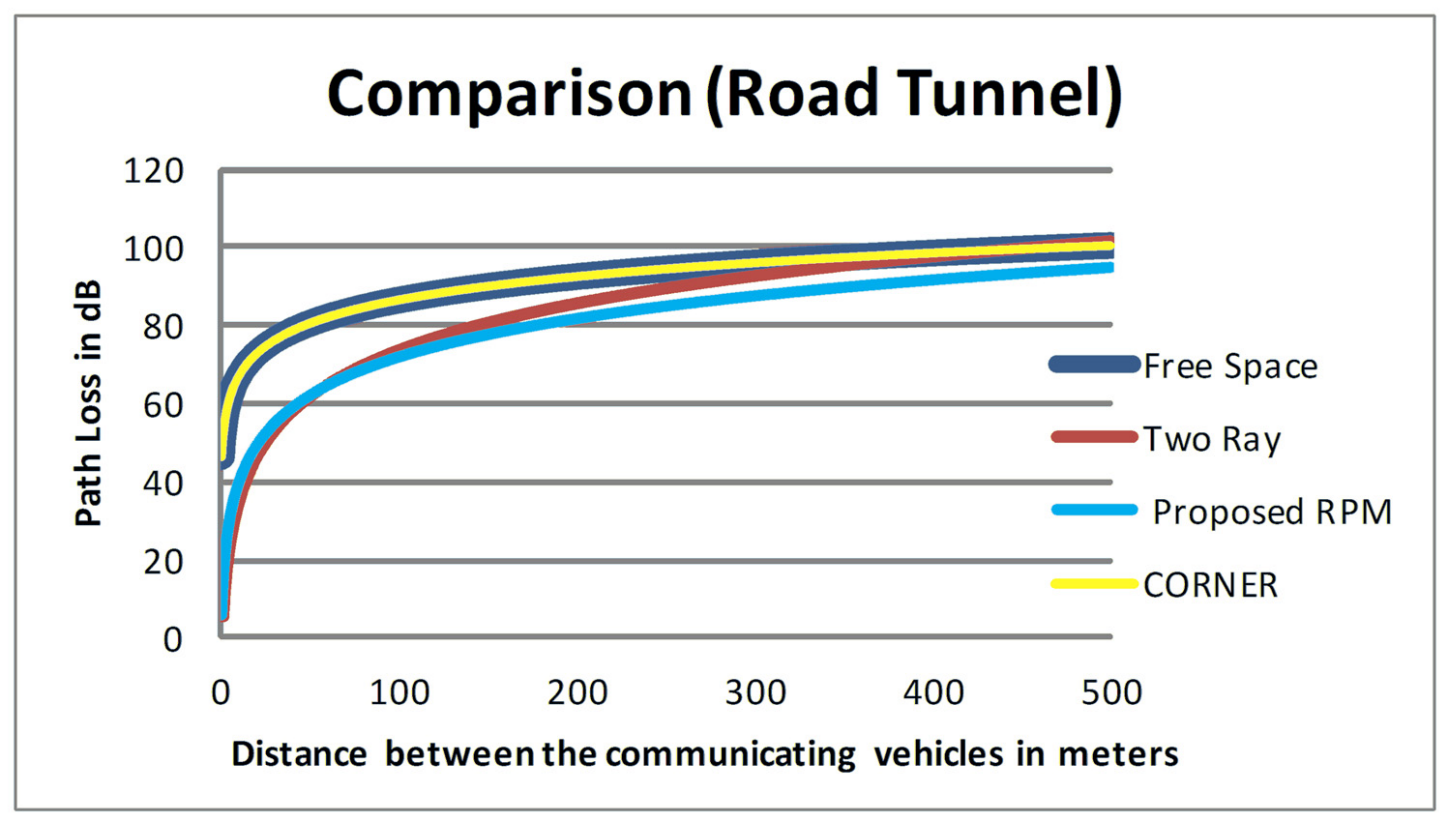
Comparison of Predicted Path Loss.

The formulas used in the proposed RPM for road tunnel are specifically designed by utilizing the tunnels dimensions and incorporating the additional attenuation caused by the moving radio obstacles to predict path loss. As we had demonstrated in this paper, the proposed RPM does not over-estimate the path loss in road tunnel, unlike existing RPMs proposed for VANETs. From our comparative summary in Fig 8, it is clear that the path loss curve resulting from application of proposed RPM in road tunnels is below all other curves. In other words, our proposed RPM better predicts the path loss as compared to existing RPMs.

A comparison among the PDR predicted by existing RPMs and the proposed RPM was performed using simulations. Simulation of Urban Mobility (SUMO) [46] was used as traffic simulator that facilitates the modelling of intermodal traffic systems. The physical sites selected for field measurements were reproduced in a simulation environment by importing the road topologies from the web-based OpenStreetMap utility into the traffic simulator SUMO. We utilized TraNSLite to convert the output of SUMO into mobility traces which are used as input in our custom-built Java Discrete Event Simulator (JaDES). JaDES was developed to scan the output from SUMO/TraNSLite and apply the proposed formulations. We utilized JaDES as a network simulation platform to simulate radio propagation in the physical layer and the results are produced in terms of RSS, path loss and PDR.

**Fig 9:**
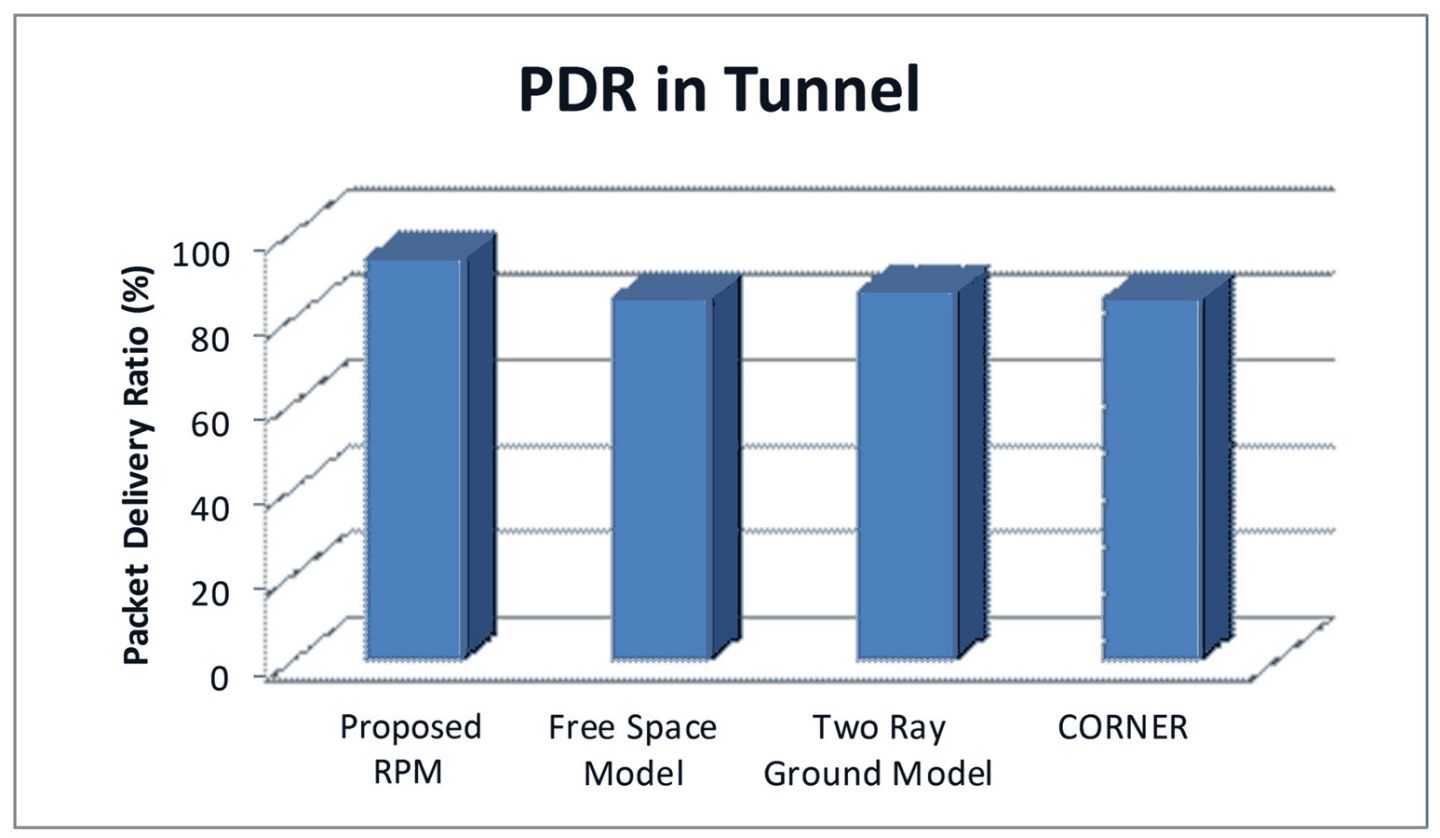
Comparison of PDR in Road Tunnel.

As shown in Fig 9, using free-space model and two-ray ground model to predict the path loss in road tunnel yielded a PDR value in the range of 85% to 87%. CORNER also models the LOS scenario as free-space model; hence, the PDR predicted by CORNER in our simulation setup is also approximately 85%. These three existing RPMs consider LOS communication with no effect of any type of radio obstacles on radio propagation. However, they ignore the positive effect of the road tunnels geometry on the signal propagation. The PDR predicted by free space model, two-ray ground model and CORNER are towards a slightly low threshold; therefore, the PDR prediction by existing RPMs is not realistic for road tunnels.

On the other hand, the proposed RPM considers the positive effects of road tunnel geometry on radio propagation. Therefore, the PDR values predicted by using the proposed RPM are in a relatively high threshold (approximately 95%) as compared to existing RPMs.

## Conclusion and Future Work

This paper presented a simple and lightweight RPM for vehicular communication in road tunnels. The proposed RPM utilizes the simple geometric characteristics of the tunnel along with the radio signal’s wavelength to predict the major component of the path loss. The impact of large moving vehicles on the radio propagation was also considered in the proposed RPM. Additional path loss component due to single knife-edge diffraction from the moving radio obstacles was added to predict the total path loss. Moreover, a field measurement campaign using 5 GHz frequency band was carried out in order to quantify the RSS and the path loss in a road tunnel. During the field measurement campaign, we used multiple setups of communicating nodes (i.e. different speeds and variable inter vehicular distances). The predicted path loss from the proposed RPM for road tunnels echoed the measured results obtained from the field measurement campaign. The proposed RPM was also compared with other widely used RPMs in VANETs.

Future work will include a more extension evaluation of the proposed model (e.g. in more physical sites and under different conditions, such as during the four seasons in New York City). This study utilized Wi-Fi devices configured at 5 GHz for field measurement. Future research will also include repeating the evaluations using 802.11p transceivers at 5.9 GHz frequency band and/or other frequency band to further affirm the suitability of the model for vehicular communication.

## Supporting Information

S1 Minimal Dataset (XLSX)
